# Activation of a GPCR, ORL1 receptor: A novel therapy to prevent heart failure progression

**DOI:** 10.21203/rs.3.rs-4578315/v1

**Published:** 2024-07-18

**Authors:** Saliha Pathan, Aarthi Pugazenthi, Beverly REA Dixon, Theodore G Wensel, Todd K Rosengart, Megumi Mathison

**Affiliations:** Baylor College of Medicine; Baylor College of Medicine; Baylor College of Medicine; Baylor College of Medicine; Baylor College of Medicine; Baylor College of Medicine

**Keywords:** Prevention of ischemic heart failure progression, ORL1 receptor activation, MCOPPB

## Abstract

**Purpose:**

The number of ischemic heart failure (HF) patients is growing dramatically worldwide. However, there are at present no preventive treatments for HF. Our previous study showed that Gata4 overexpression improved cardiac function after myocardial infarction in the rat heart. We also found that Gata4 overexpression significantly increased a Pnoc gene expression, an endogenous ligand for cell membrane receptor, ORL1. We hypothesized that an activation of ORL1 receptor would suppress HF in a rat ischemic heart model.

**Method:**

Adult Sprague Dawley rats (8 weeks old, 6 males and 6 females) underwent left anterior descending coronary artery ligation. Three weeks later, normal saline or MCOPPB (ORL1 activator, 2.5mg/kg/day) intraperitoneal injection was started, and continued 5 days a week, for 3 months. Echocardiography was performed six times, pre-operative, 3 days after coronary artery ligation, pre-MCOPPB or saline injection, and 1, 2, and 3 months after saline or MCOPPB injection started. Animals were euthanized after 3 months follow up and the heart was harvested for histological analysis.

**Results:**

ORL1 activator, MCOPPB, significantly improved cardiac function after myocardial infarction in rat (Ejection fraction, MCOPPB vs saline at euthanasia, 67 ± 3 vs 43 ± 2, p < 0.001). MCOPPB also decreased fibrosis and induced angiogenesis.

**Conclusion:**

ORL1 activator, MCOPPB, may be a novel treatment for preventing HF progression.

## Introduction

During the past two decades, we and several other researchers, reported that Gata4 overexpression (OE) improved cardiac function after myocardial infarction (MI) in the murine heart [1–3]. Dr. Ruskoaho’s group [4] also reported that Gata4 reduced angiotensin-induced remodeling in rat hearts by interfering with pro-fibrotic and hypertrophic gene expression. Despite those positive reports, Gata4 OE has not yet been pursued as a HF treatment. Since Gata4 is an essential transcription factor in gene regulation of cardiac hypertrophy, recent research has been focused on targeting Gata4 as a modulator of post-translational modification [5].

**Figure 1:**
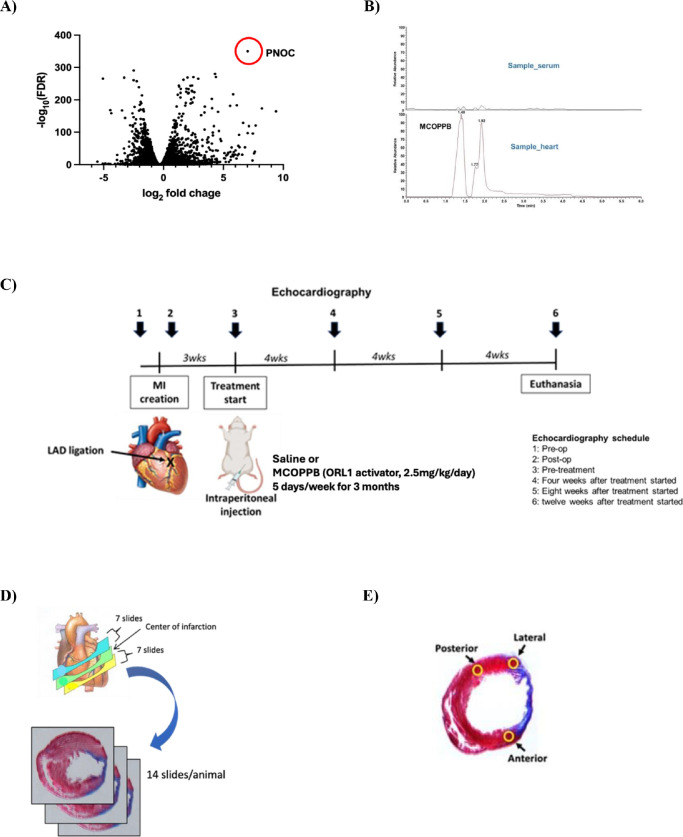
Volcano plot of RNAseq analysis. **A)** Cardiac fibroblasts were treated with lentivirus encoding Gata4 or GFP (each 20MOI) for 14 days (n=3). PNOC gene was significantly upregulated by Gata4. **B) Chromatography assay for MCOPPB detection in the heart.** Rats received MCOPPB 2.5mg/kg/day or saline intraperitoneally for 4 days (n=3/group) and were euthanized immediately after the last injection. The heart tissues were examined with Mass spectrometry. MCOPPB was detected in the heart tissue of all MCOPPB received rats but no MCOPPB in the saline received rats. Representative chromatography of MCOPPB received rat is shown. **C) Schematic showing the experimental design of MCOPPB study.** Twelve adult Sprague Dawley rats were enrolled. Three weeks after the coronary artery ligation, they were treated with saline or MCOPPB for 3 months; six rats (3 males and 3 females) for saline, and another 6 rats (3 males and 3 females) for MCOPPB. Echocardiography was performed six times, 1: Pre-op, 2: Post-op, 3: Pre-treatment, 4: Four weeks after treatment started, 5: Eight weeks after treatment started, and 6: twelve weeks after treatment started. **D) Fibrosis analysis.** The excised heart was cut transversally and sectioned with 2 (2- to 3-mm thick) slices obtained, One, immediately cephalad and another one immediately caudad to the transverse centerline of the infarct region, which was readily identifiable by gross inspection. To assess the extent of fibrosis, 14 sections per animal (at a 120-μm interval between each section) were stained with Masson’s trichrome. **E) Three regions where cardiomyocyte diameter was measured:** The slide demonstrating the greatest area of fibrosis, as identified by Masson’s Trichrome staining, was selected for each animal. In each slide, 20 longitudinally oriented cardiomyocytes from each 3 regions, anterior, lateral, and posterior, were examined, and the diameters were defined. The mean value of 20 measurements represented 1 sample from each position in each animal.

After we reported that Gata4 OE suppressed HF [3], we further investigated the effects of Gata4 OE *in vitro*. Our RNAseq analysis revealed that the Pnoc gene was significantly upregulated by Gata4 OE in cardiac fibroblasts (Fig. 1A). Pnoc is a preproprotein and is proteolytically processed to generate nociceptin, which binds to ORL1. ORL1 belongs to the G protein-coupled receptor (GPCR) and is the most recently discovered member of the opioid receptor family [6]. Pnoc and ORL1 have been intensely investigated in the neuroscience field [6]. However, it has heretofore not been investigated in HF research.

The aim of this study was to investigate if activation of ORL1 inhibits HF progression.

## Material and methods

### Animals

All animal experiments were performed in accordance with IACUC protocol approved by Baylor College of Medicine (no. AN-6223). Animals were housed and cared for in facilities run by the Center for Comparative Medicine at Baylor College of Medicine, which is fully accredited by the Association for the Assessment and Accreditation of Laboratory Animal Care International. Adult Sprague Dawley rats (Inotiv co.) (8 weeks old, 6 males and 6 females) underwent left anterior descending (LAD) coronary artery ligation (described in detail in our previous publication [7]). In brief, animals were first anesthetized with isofluorane 4% in an induction box, intubated, and placed on a rodent ventilator (Harvard Apparatus) using isofluorane inhalation (3%) supplemented with oxygen. A left thoracotomy was then performed, and the left coronary artery was ligated 1 to 2 mm from its origin with a 7–0 polypropylene suture.

### ORL1 activator

We selected MCOPPB (Sigma-Aldrich, PZ0159) as an ORL1 receptor activator. MCOPPB is reported as the most potent and novel non-peptide ORL1 full agonist drug *in vitro*.^5^ [8] Dosage of MCOPPB of 2.5mg/kg/day was chosen based on the article published by Raffaele M et al. [9] They administered MCOPPB of 5mg/kg /day to mice by intraperitoneal injection. According to the guide for dose conversion between animals [10], we administered MCOPPB of 2.5mg/kg/day to the rats. In order to confirm that MCOPPB is delivered to the heart with this dosage, we tested MCOPPB of 2.5mg/kg/day or normal saline administration intraperitoneally in 3 rats/group for 4 days after coronary artery ligation and the heart was harvested. Mass spectrometry confirmed MCOPPB upregulation in the heart of MCOPPB received rats, but no MCOPPB upregulation was observed in the heart of saline received rats (Fig. 1B).

### Treatment

Three weeks after the coronary ligation, normal saline or MCOPPB (2.5mg/kg/day) intraperitoneal injection was started, and continued 5 days per week, for 3 months (Fig. 1C).

### Echocardiography

Echocardiography (Veno 770 Imaging System, VisualSonics) was performed six times, pre-operative, 3 days after coronary artery ligation, pre-MCOPPB or saline injection, and 1, 2, and 3 months after saline or MCOPPB injection started (Fig. 1C). Echo images of the left ventricle were obtained in short-axis views by investigators blinded to treatment group. Left ventricular end-systolic and end-diastolic diameters and left ventricular septal and posterior thickness (in both end-systolic and end-diastolic phases) were measured from M-mode tracings. These imaging data were then analyzed by investigators blinded to treatment group.

### Euthanasia and histological analysis

Animals were euthanized after 3 months follow up and the heart was harvested. For the histological analysis, the excised heart was cut transversally and sectioned with 2 (2- to 3-mm) slices obtained, one, immediately cephalad and the other one immediately caudad to the transverse centerline of the infarct region, which was readily identifiable by gross inspection (Fig. 1D). To assess the extent of fibrosis, 14 sections per animal (at a 120-μm interval between each section) were stained with Masson’s trichrome (Fig. 1D). The fibrotic area (blue) and the nonfibrotic region (red) were outlined using Adobe Photoshop CS5 software, and then quantified with MATLAB and Simulink software (MathWorks, Inc). The percent fibrosis was calculated as: (Total of blue pixels from all sections / Total of blue plus red pixels from all sections) × 100.

Cardiomyocyte diameter was measured at 400x magnification of cardiomyocytes found in the peri-infarct (anterior, lateral) regions subtended by the ligated left anterior descending coronary artery and the non-infarcted (posterior) LV regions (Fig. 1E). The slide demonstrating the greatest area of fibrosis, as identified by Masson’s Trichrome staining, was selected for each animal. In each slide, 20 longitudinally oriented cardiomyocytes from each 3 regions, anterior, lateral, and posterior, were examined, and the diameters were defined. The mean value of 20 measurements represented 1 sample from each position in each animal.

For angiogenesis analysis, two sections per animal, in which infarction size was largest in the transverse section, were stained with CD31 (R&D systems, AF3628). First, the stained sections were searched for CD31-positive cells by five random microscopic fields per slide at x200 magnification and the highest number identified in peri-infarct region was chosen as a number of CD31 positive cell for each slide.

### Randomization and blinding

Rats were randomly allocated to the experimental groups, 3 males and 3 females to each group, saline and MCOPPB. All experiments, including animal surgery, echocardiography, and echocardiographic and histological analyses were conducted by researchers blinded to treatment group.

### Statistical analysis

Statistical analysis was performed with SAS version 9.4. The data were presented as mean ± SD. ANOVA was performed to detect statistical significances between multiple groups. When the ANOVA showed significance, a Two-tailed ANOVA with Bonferroni post hoc test was performed. Values of *P* < 0.05 were considered statistically significant.

## Results

**Figure 2:**
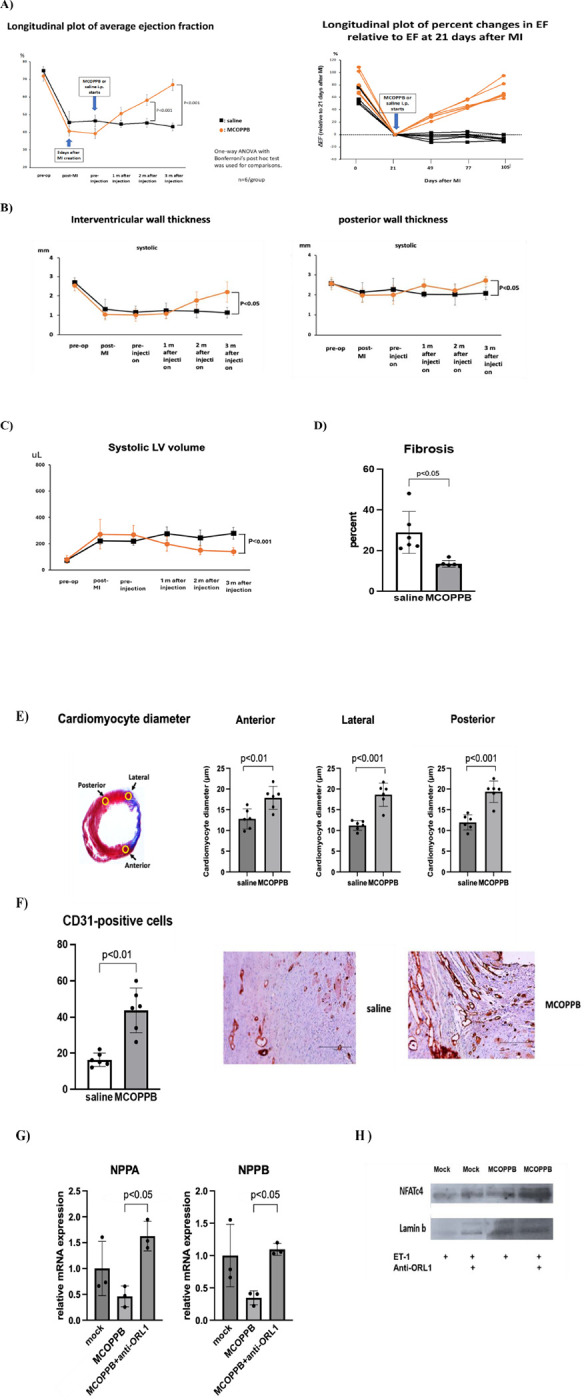
Cardiac function was improved with MCOPPB administration. **A)** Echocardiography results show longitudinal plot of changes in average ejection fraction (graph on left) and individual relative percent changes in ejection fraction (graph on right) associated with saline (black line) and MCOPPB (orange line) administration, started at day 21 after myocardial infarction induction. Ejection fraction significantly improved in the MCOPPB group (MCOPPB vs saline at 2 months follow-up, 58±3 vs 45±2, p<0.001, MCOPPB vs saline at euthanasia, 67±3 vs 43±2, p<0.001). Two-tailed ANOVA with Bonferroni post hoc test was used. n=6/group. **B) Systolic wall thickness increased in MCOPPB group.** LV wall thickness was measured by echocardiography with short axis M-mode image, at interventricular wall (left), and posterior wall (right). Both end-systolic interventricular septum (IVS) (left) and end-systolic left ventricular posterior wall (LVPW) (right) were significantly greater in MCOPPB group compared to saline group at euthanasia (end-systolic IVS, MCOPPB vs saline, 2.2±0.5mm vs 1.1±0.3mm, p<0.01, end-systolic LVPW, MCOPPB vs saline, 2.7±0.2mm vs 2.1±0.3mm, p<0.05). Two-tailed ANOVA with Bonferroni post hoc test was applied. n=6/group. Orange line: MCOPPB group, black line : saline group. **C) End-systolic volume was significantly decreased in MCOPPB-received group at euthanasia.** End-systolic volume was calculated with VenoLAB software: (7.0/(2.4+LVIDs))xLVIDs^3^, LVIDs=left ventricular internal diameter end systole). End-systolic volume was significantly decreased in MCOPPB-received group at euthanasia (MCOPPB vs saline, 140±30μL vs 280±44 μL, p<0.001). Orange line: MCOPPB group, Black line: Saline group. Two-tailed ANOVA with Bonferroni post-hoc test was used. n=6/group. **D) MCOPPB decreased extent of left ventricular wall fibrosis.** The percent of left ventricular wall fibrosis was determined by Masson-Trichrome staining of the sections of myocardial tissue harvested 3 months after the administration of MCOPPB or saline (Figure 1D). Fibrosis area significantly decreased in MCOPPB group (% fibrosis area, MCOPPB vs saline, 14±2 vs 29±10, p<0.05). Two-tailed t-test was used. **F) MCOPPB increased angiogenesis in infarcted heart.** Myocardial angiogenesis was analyzed by immunohistochemical staining with CD31 antibody. The number of capillaries was counted with two slides per animal, most positive cells found in peri-infarct region (x200 magnification). MCOPPB group had significantly higher vessel counts in border zone (44±12 vs 16±4, p<0.01). The graph shows the number of vessels/field. n=6/group. Two-tailed t test was used. Photo-images are two representative slides from saline and MCOPPB groups. Scale bar=150μm. **G) NPPA and NPPB were downregulated by MCOPPB.** Rat neonatal cardiomyocytes were treated with nociception agonist, MCOPPB (0.5mM), ET-1 (100nM) and anti-ORL1(ORL1 antagonist, [Nphe1]Nociceptin(1–13)NH2]) (10mM). qPCR shows that NPPA and NPPB, which are downstream transcription genes of NFAT signaling pathway and related to pathological hypertrophy, were downregulated by MCOPPB. Significantly, those downregulations by MCOPPB were diminished by adding ORL1 antagonist. Two-tailed ANOVA with Bonferroni post hoc test was used. Primers for NPPA: forward, 5’-CGTATACAGTGCGGTGTCCAAC-3’. reverse, 5’-CATCTTCTCCTCCAGGTGGTCTAG-3’. Primers for NPPB: forward, 5’-AAGTCCTAGCCAGTCTCCAGAACA −3’. Reverse, 5’-TTGAGAGCTGTCTCTGAGCCATT-3’. **H) ORL1 inhibitor increased nuclear dephosphorylated NFAT.** Rat neonatal cardiomyocytes were treated with MCOPPB (0.5 μM), ET-1 (100nM) and anti-ORL1 (10 μM). Nucler fractions of cells were extracted using NE-PER nuclear reagents kit and analyzed by western blotting with NFATc4 antibody(Santa Cruz Biotechnology- SC 271597). Lamin b (Santa Cruz Biotechnology- SC 374015) was used as a loading control. NFATc4 expression was significantly increased by adding ORL1 antagonist.

None of the animals showed any notable side effects during the follow-up period. Echocardiographic data showed that ejection fraction significantly improved in the MCOPPB group (MCOPPB vs saline at 2 months follow-up, 58 ± 3 vs 45 ± 2, p < 0.001, MCOPPB vs saline at euthanasia, 67 ± 3 vs 43 ± 2, p < 0.001) (Fig. 2A). Further, measurement of wall thickness by echocardiography showed that both end-systolic left ventricular posterior wall (LVPW) and end-systolic interventricular septum (IVS) were significantly greater in MCOPPB group compared to saline group at euthanasia (end-systolic LVPW, MCOPPB vs saline, 2.7 ± 0.2mm vs 2.1 ± 0.3mm, p < 0.05, end-systolic IVS, MCOPPB vs saline, 2.2 ± 0.5mm vs 1.1 ± 0.3mm, p < 0.01) (Fig. 2B), and end-systolic volume was significantly decreased in MCOPPB-received group at euthanasia (MCOPPB vs saline, 140 ± 30μL vs 280 ± 44 μL, p < 0.001) (Fig. 2C). Fibrosis area, stained by Masson-Trichrome, significantly decreased in MCOPPB group (% fibrosis area, MCOPPB vs saline, 14 ± 2 vs 29 ± 10, p < 0.05) (Fig. 2D). Cardiomyocyte diameter was significantly increased in MCOPPB-received group (MCOPPB vs saline; anterior, 18 ± 3μm vs 13 ± 2μm, p < 0.01, lateral, 19 ± 3μm vs 11 ± 1μm, p < 0.001, posterior, 19 ± 3μm vs 12 ± 2μm, p < 0.001) (Fig. 2E). Angiogenesis was assessed by CD31 staining and MCOPPB group had significantly higher vessel counts in border zone (44 ± 12 vs 16 ± 4, p < 0.01) (Fig. 2F).

## Discussion

Our *in vivo* experiments demonstrated that MCOPPB alone without Gata4 overexpression, improved cardiac function, suppressed fibrosis, and induced angiogenesis, resulting in attenuating HF. In the echocardiographic analysis, there were three significant data points; 1) ejection fraction improved, 2) end-systolic volume is decreased, and 3) end-systolic wall thickness is increased. 1) and 2) suggest increased cardiac contractility. 3) suggests cardiac hypertrophy. Also, immunohistochemistry analysis showed that cardiomyocyte diameter is increased. Since cardiac function is improved, we believe that the hypertrophy is not pathological, but rather physiological. We recently performed preliminary *in vitro* experiments and we found that MCOPPB downregulated pathological hypertrophy-related genes, NPPA and NPPB [11](Fig. 2G). We also observed that nuclear NFAT was greatly increased by administering ORL1 inhibitor (Fig. 2H). This suggests that Calcineurin-NFAT pathway, which is an up-stream signaling pathway of NPPA and NPPB [11], is downregulated by MCOPPB.

Our next question is, “If MCOPPB downregulates pathological hypertrophy pathway, does MCOPPB upregulate physiological hypertrophy pathway?”. GPCRs are known to be a co-activator of receptor tyrosine kinase (RTK) [12]. Physiological hypertrophy inducer, IGF receptor, belongs to RTK. Therefore, the ORL1 receptor, activated by MCOPPB, might co-activate the IGF receptor, which induces physiological hypertrophy. The VEGF receptor also belongs to RTK, and activated VEGF receptor induces angiogenesis. Our finding that MCOPPB induced angiogenesis also might be a result of co-activation of VEGF receptor by MCOPPB.

Our goal is to translate MCOPPB treatment into clinically effective and safe therapy for combatting HF progression. Our *in vivo* experiment demonstrated that post-MI rats did not show any notable side effects with MCOPPB intraperitoneal injection for 3 months. Raffaele M et al. [9] administered MCOPPB (5mg/kg/day) intraperitoneally to mice for 2 months to investigate if MCOPPB has anxiolytic and senolytic effects. They observed weight gain and mild hepatic stress characterized by a low grade of steatosis. In our study, we did not examine the liver. Regarding body weight, no significant weight gain in the MCOPPB group was observed compared to saline group (% body weight gain; Saline group vs MCOPPB group: 15±8% vs 13±8%). We plan to perform further experiments with prolonged administration of MCOPPB as well as increased animal numbers in order to evaluate any side effects. Further, we plan to perform efficacy and safety studies with MI-induced swine since pigs are widely accepted as a clinically relevant model in cardiac research. We plan to treat pigs with MCOPPB via an osmotic pump embedded in the neck subcutaneous area. Since MCOPPB can be orally administered [13], we will also investigate the effectiveness of oral administration.

### Study limitation

The preliminary *in vitro* experiments suggest underlying molecular mechanisms that enables MCOPPB to suppress HF. However, the experiments are incomplete. We plan to repeat thorough *in vitro* experiments. We expect that further studies on the molecular mechanism of MCOPPB can also elucidate potential wanted and unwanted effects of its use.

## Data Availability

Original data will be available upon request.
